# 4-Amino­benzoic acid 4-methyl­pyridine/4-methyl­pyridinium 4-amino­benzoate 0.58/0.42: a redetermination from the original data

**DOI:** 10.1107/S2056989017013226

**Published:** 2017-09-19

**Authors:** Jan Fábry

**Affiliations:** a Inst. of Physics of the Czech Academy of Sciences, Na Slovance 2, 182 21 Praha 8, Czech Republic

**Keywords:** crystal structure, redetermination, hydrogen bonding, symmetric hydrogen bonds, refinement constraints

## Abstract

The title structure, 4-amino­benzoic acid 4-methyl­pyridine/4-methyl­pyridinium 4-amino­benzoate 0.58/0.42, 0.58(C_6_H_7_N·C_7_H_7_NO_2_)·0.42(C_6_H_8_N^+^·C_7_H_6_NO_2_^−^), has been redetermined from the data published by Kumar *et al.* (2015 ▸). *Acta Cryst.* E**71**, o125-o126. The improvement of the present redetermination consists in the introduction of disorder of the methyl group over two positions as well as in the correction of the positional parameters of the hydrogen atoms that are involved in the O—H⋯N or N—H⋯O hydrogen bonds. After the correction, the hydroxyl hydrogen atom turned out to be disordered over two positions about the centre of the O⋯N bond, which is relatively long [2.642 (2) Å], while the H atoms of the primary amine group account more realistically for the hydrogen-bond pattern after the removal of the positional constraints. All the O—H⋯N or N—H⋯O hydrogen bonds which are present in the title structure are of moderate strength.

## Abstract 2

The title structure, 4-amino­benzoic acid 4-methyl­pyridine/4-methyl­pyridinium 4-amino­benzoate 0.58/0.42, has been redetermined from the data published by Kumar *et al.* (2015). *Acta Cryst.* E**71**, o125-o126. The motivation for this redetermination follows from negligence of important features of the difference electron-density maps as well as from wrongly applied constraints, which significantly affect the structural model. The corrections affect mainly the positions of the H atoms involved in the hydrogen bonds (centered on the primary amine group for which the H atom turned out to be disordered over two positions about the centre of an N⋯H⋯O hydrogen bond) and the methyl group, which is disordered and has now been remodelled.

## Chemical context   

**Figure 1:**
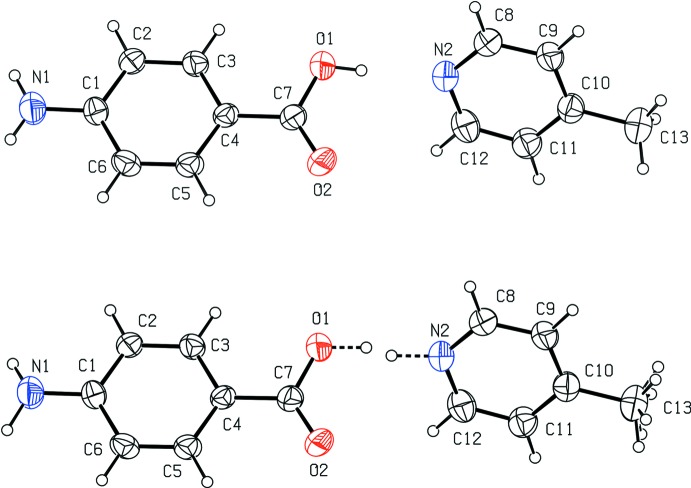
View of the constituent mol­ecules of the title structure (top: the original determination (Kumar *et al.*, 2015 ▸); bottom: present redetermination). Displacement ellipsoids are depicted at the 50% probability level.

Crystal structures that contain hydroxyl, secondary and primary amine groups are sometimes determined incorrectly because of an assumed geometry of these groups from which the applied constraints or restraints were inferred. In such cases, the correct geometry is missed as it is not verified by inspection of the difference electron-density maps. Thus a considerable number of structures could have been determined more correctly – *cf.* Figs. 1 ▸ and 2 ▸ in Fábry *et al.* (2014 ▸). The inclusion of such structures causes bias in crystallographic databases such as the Cambridge Crystallographic Database (CSD; Groom *et al.*, 2016 ▸).
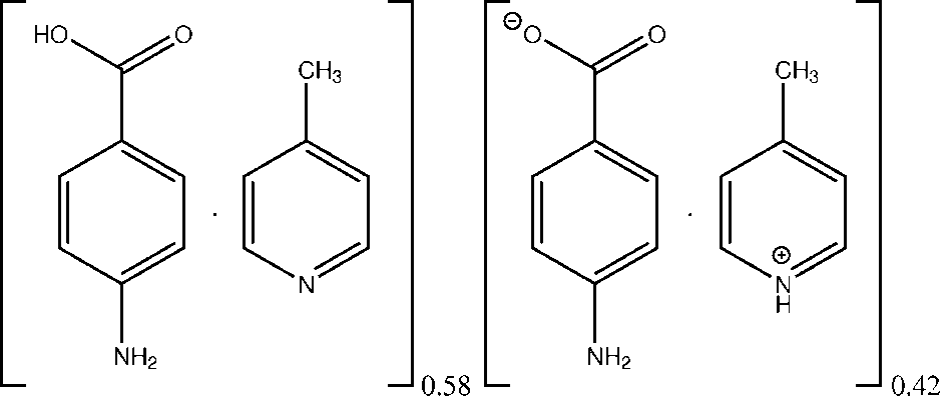


In the course of recalculation of suspect structures that were retrieved from the CSD, the structure determination of the title structure by Kumar *et al.* (2015 ▸), CSD refcode WOYPEH, became a candidate for a checking recalculation. The reason was that the primary amine group centered on N1 was constrained to be coplanar to the attached phenyl group with distances N1—H1*a* and N1—H1*b* constrained to be equal to 0.86 Å with *U*_iso_(H_primary/secondary amine_) = 1.2*U*_eq_(N_primary/secondary amine_).

The hydroxyl hydrogen atom H1 was also suspect because the O—H bond length was reported to be restrained to the value 0.82 Å [the estimated standard deviation/elasticity (Müller *et al.*, 2006 ▸) was not given in the original article]. However, the distance reported by Kumar *et al.* (2015 ▸) is 0.836 (10) Å, which indicated that the bridging hydrogen atom might have been situated towards the centre of the pertinent O1⋯N2 hydrogen bond. Recalculation with *JANA*2006 (Petříček *et al.*, 2014 ▸) revealed hydrogen atom H1 to be disordered over two positions about the centre of the O1⋯N1 hydrogen bond in almost equal proportions, 0.58(*7*) (H1*x*) and 0.42(*7*) (H1*y*) (Figs. 1 ▸ and 2 ▸). This is different from the situation reported in the original article (Kumar *et al.*, 2015 ▸). Moreover, inspection of the difference electron-density maps has also revealed quite a smeared electron density pertinent to the methyl hydrogen atoms (Fig. 3 ▸).

## Structural commentary   

**Figure 2:**
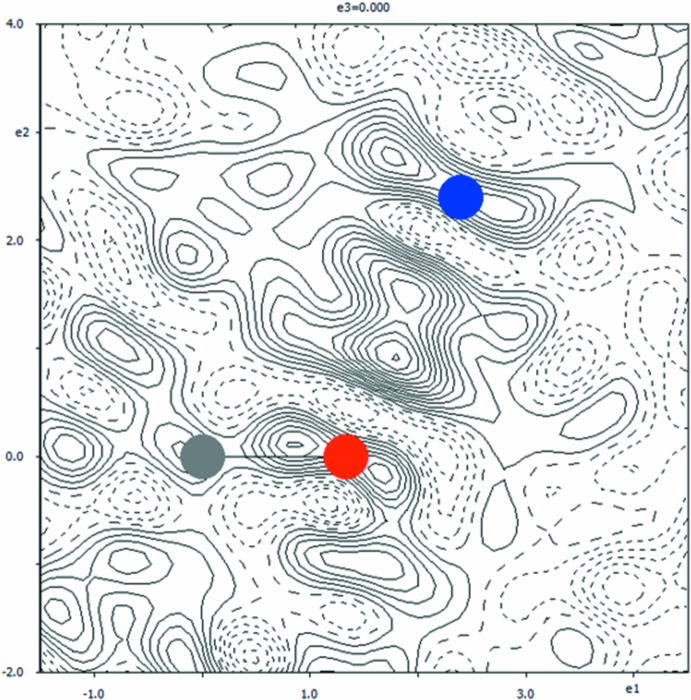
A section of the difference electron-density map for the redetermined title structure without the atoms H1*x* and H1*y*. A build-up of the electron density between the atom O1 (red) and N2^iii^ (blue) [symmetry codes: (iii) *x* + 1, *y*, *z* + 1] is shown; the larger and the smaller peaks correspond to the electron density of 0.12 and 0.11 e A^−3^, respectively. These peaks were assigned to the respective positions of H1*x* and H1*y* ^iii^. The positive and negative electron densities are indicated by continuous and dashed lines, respectively. The increment of the electron density between neighbouring contours is 0.01 e Å^−3^. Atom C7 is indicated by a gray circle.

Table 1 ▸ lists the hydrogen bonds in the structure which are shown in Fig. 4 ▸. All the hydrogen bonds are of moderate strength (Gilli & Gilli, 2009 ▸). However, the hydrogen bond O1—H1*x*⋯N2^iii^/O1⋯H1*y* ^iii^—N2^iii^ [2.642 (2) Å; symmetry code: (iii) *x* + 1, *y*, *z* + 1] is quite long for an O⋯N hydrogen bond with a disordered bridging hydrogen atom, *i.e.* for a hydrogen atom the substantial part of its electron density is situated along the connecting line between the donor/acceptor atoms as happens in O1—H1*x*⋯N2^iii^/O1⋯H1*y* ^iii^—N2^iii^ of the title structure (Fig. 2 ▸). This O1⋯N2^iii^ hydrogen bond is even longer than the O3⋯N1 hydrogen bond with a disordered bridging hydrogen atom that was observed in a recently determined structure 2,4,6-tri­amino­pyrimidinium(1+)_*x*_ hydrogen trioxo­fluoro­phosphate(1−)_*x*_ monohydrate/2,4,6-tri­amino­pyrimidinium(2+)_(1–*x*)_ trioxo­fluoro­phosphate(2−)_(1–*x*)_ monohydrate, where *x* = 0.73, at room temperature (Matulková *et al.*, 2017 ▸). The latter O⋯N hydrogen bond measured to be 2.5822 (16) Å and is ranked among the longest known O⋯N hydrogen bonds with a disordered bridging hydrogen atom.

On the other hand, the tendency for a hydrogen atom to be situated just between the donor and acceptor atoms has been observed for strong hydrogen bonds, especially of the type O⋯H⋯O (Gilli & Gilli, 2009 ▸). Such bonds tend to occur in the structures where the difference Δp*K*_a_ = p*K*_a_(base) − p*K*_a_(acid) is close to 0 (Gilli *et al.*, 2009 ▸). The difference Δp*K*_a_ is correlated with the occurrence of structures where the base and acid components are not ionized, thus forming a co-crystal (Δp*K*_a_ < 0), or ionized, forming a salt (Δp*K*_a_ > 3; Childs *et al.*, 2007 ▸). It is difficult to predict the form in which the acid and the base are present for 0 < Δ*p*K_a_ < 3.

In the case of the title structure, p*K*_a_ of 4-methyl­pyridine and of 4-amino­benzoic acid are equal to 5.99 (CRC Handbook of Chemistry and Physics, 2009 ▸) and 2.38 (Kortüm *et al.*, 1961 ▸), respectively. Thus Δp*K*_a_ = 3.61 for the title structure, which means that the salt form should be slightly more probable for the present structure.

The primary amine group centered on N1 was originally constrained to be coplanar with the attached phenyl ring while the N1—H1*a* and N1—H1*b* distances were both constrained to 0.86 Å.

The difference electron-density map in the plane of the methyl hydrogen atoms that were excluded from the structure for the sake of this checking calculation (Fig. 4 ▸) shows that the methyl group can be better modelled by a disorder over two positions with equal occupancies. The disordered positions of the methyl group are related by a rotation of 60.19 (5)° about the C10—C13 bond.

Table 1 ▸, which also compares the values of the hydrogen-bond pattern in the title and the original structures (Kumar *et al.*, 2015 ▸), emphasizes the importance of a careful examination of the difference electron-density maps during structure determinations. It serves as an example of the bias that is caused by unsubstanti­ated constraints of the primary amine groups as well as by constraints or restraints imposed on the hydroxyl groups.

## Supra­molecular features   

The strongest hydrogen bond O1—H1*x*⋯N2^iii^/O1⋯H1*y* ^iii^—N2^iii^; symmetry code: (iii) *x* + 1, *y*, *z* + 1] with a bridging hydrogen atom disordered over two positions (H1*x* and H1*y* ^iii^) forms a finite *D*(3) pattern (Etter *et al.*, 1990 ▸) on a local scale (Figs. 1 ▸ and 4 ▸).

The primary amine group, which is centered on atom N2, is involved in the hydrogen-bond pattern with a pair of symmetry-equivalent O2 atoms. It forms an 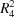
(20) graph-set motif, shown in Fig. 4 ▸, in which two 4-amino­benzoic acid/amino­benzoate mol­ecules with the symmetry codes (i) and (ii) are involved [symmetry codes: (i) −*x* + 1, *y*, *z* − 1; (ii) −*x* + 1, −*y*, *z* − 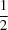] as well as the atoms of the primary amine groups H1*a-*-N1–H1*b* and atom O2^iv^ [symmetry code: (iv) −*x* + 2, −*y*, *z* − 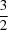].

## Database survey   

The structure determination by Kumar *et al.* (2015 ▸) is included in the Cambridge Structural Database (Groom *et al.*, 2016 ▸) under refcode WOYPEH.

## Synthesis and crystallization   

The preparation of the title crystals was described by Kumar *et al.* (2015 ▸).

## Refinement   

Table 2 ▸ lists the details regarding the crystal data, data collection and the refinement [some pieces of information were taken from the downloaded CIF of the original article by Kumar *et al.* (2015 ▸)]. The refinement was carried out on the data for which the 826 Friedel pairs were not merged. Since the structure is composed of light atoms only and the applied radiation was Mo *K*α the absolute structure could not be determined.

All hydrogen atoms were discernible in the difference electron-density map. The aryl hydrogens were constrained by the constraints C_ar­yl_—H_ar­yl_ = 0.93 Å and *U*_iso_(H_ar­yl_) = 1.2*U*_eq_(C_ar­yl_). The positional parameters of the primary amine hydrogen atoms H1*a* and H1*b* were refined freely while their displacement parameters were constrained by *U*_iso_(H_N1_) = 1.2*U*_eq_(N1).

The positional parameters of the bridging hydrogen atoms, H1*x* and H1*y*, were determined from difference electron-density maps (Fig. 2 ▸) and fixed in the subsequent refinement. Their isotropic displacement parameters were set equal and their occupational parameters were refined under the condition that the sum of their occupancies was equal to 1.

**Figure 3:**
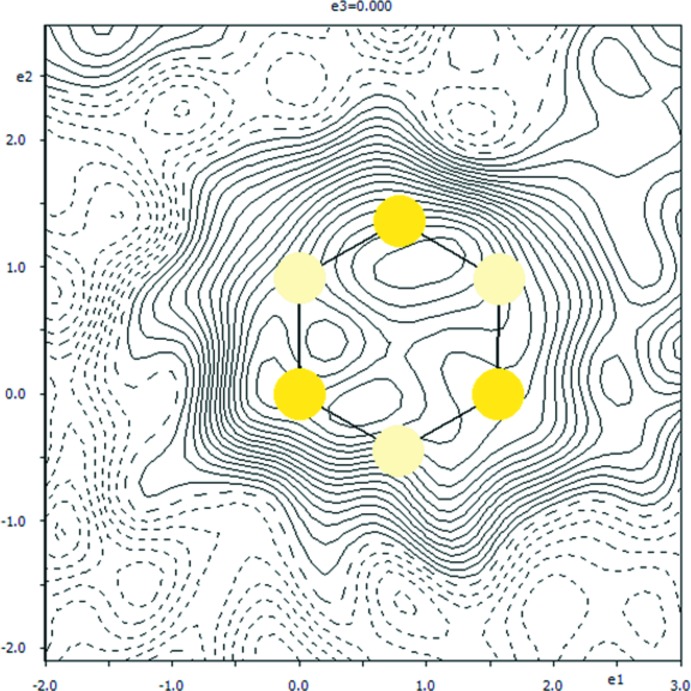
A section of the difference electron-density map for the redetermined title structure without the methyl H atoms. The positions of both methyl-hydrogen triplets are indicated by yellow circles of a different hue. The positive and negative electron densities are indicated by continuous and dashed lines, respectively. The increment of electron density between the neighbouring contours is 0.01 e Å^−3^.

**Figure 4:**
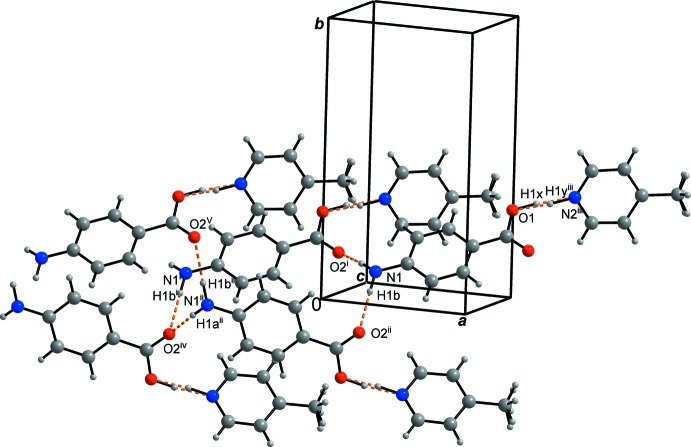
A section of the title structure. Symmetry codes (i): −*x* + 1, *y*, *z* − 1; (ii): −*x* + 1, −*y*, *z* − 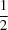; (iii): *x* + 1, *y*, *z* + 1; (iv): −*x* + 2, −*y*, *z* − 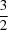 (v): *x* − 2, *y*, *z* − 1. Applied colours for the atoms: grey – C and H, blue – N, O – red; applied colours for the bonds: black – covalent bonds, dashed orange – hydrogen bonds.

The electron density in the plane of the methyl hydrogen atoms, which was centered on atom C13, was found to be quite smeared (Fig. 3 ▸). It was modelled by a disorder over two positions with equal occupancies. The rotation between both triplets of the methyl hydrogen atoms is 60.19 (5)°. In order to account for this model, dummy atoms C10*a* and C13*a*, both with occupancies equal to 0, were introduced into the structure; their atomic parameters were otherwise constrained to be equal to those of atoms C10 and C13, respectively. The methyl hydrogen atoms were constrained by distance constraints C_meth­yl_—H_meth­yl_ = 0.96 Å with *U*_iso_(H_meth­yl_) = 1.5*U*_eq_(C_meth­yl_).

It is worthwhile mentioning that the recalculation of the original model with *JANA*2006 (Petříček *et al.*, 2014 ▸) in order to reproduce the original constraints and restraints converged with difficulty {Δ[last step of the parameter(*i*)]/σ(*i*) < 0.6}. The indicators of the refinement of such a model were substanti­ally higher: *R*_obs_ = 0.0503, *Rw*_obs_ = 0.1035, *R*_all_ = 0.0930, *Rw*_all_ = 0.1119. The condition for the observed diffractions was *I*/σ(*I*) > 3, *cf*. Table 2 ▸ for indicators of the refinement for the redetermined structure.

## Supplementary Material

Crystal structure: contains datablock(s) global, I. DOI: 10.1107/S2056989017013226/su5390sup1.cif

Structure factors: contains datablock(s) I. DOI: 10.1107/S2056989017013226/su5390Isup2.hkl

Supporting information file. DOI: 10.1107/S2056989017013226/su5390Isup3.cml

CCDC reference: 1574686

Additional supporting information:  crystallographic information; 3D view; checkCIF report

## Figures and Tables

**Table 1:** Hydrogen bonds (Å, °) in the redetermined structure as well as in the determintion by Kumar *et al.* (2015 ▸). Some of the atoms in the original article were transformed

Bond	*D*—H	H⋯*A*	*D*⋯*A*	*D*—H⋯*A*
This determination:				
N1—H1*a*⋯O2^i^	0.88 (2)	2.22 (3)	3.051 (3)	158 (3)
N1—H1*b*⋯O2^ii^	0.99 (3)	2.04 (3)	3.028 (3)	179 (2)
O1—H1*x*⋯N2^iii^	1.0154 (14)	1.6303 (18)	2.642 (2)	173.65 (11)
N2—H1*y*⋯O1^i^	1.0719 (18)	1.5740 (14)	2.642 (2)	173.55 (12)
				
Determination by				
Kumar *et al.* (2015 ▸):				
N1—H1*a*⋯O2^i^	0.86	2.32	3.049 (3)	142
N1—H1*b*⋯O2^ii^	0.86	2.17	3.031 (3)	174
O1—H1⋯N2^ii^	0.84 (1)	1.81 (1)	2.644 (3)	177 (4)

Symmetry codes: (i) *x* − 1, *y*, *z* − 1; (ii) *x* − 1, −*y*, *z* − 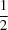; (iii) *x* + 1, *y*, *z* + 1.

**Table 2:** Experimental details

Crystal data	
Chemical formula	0.58(C_6_H_7_N·C_7_H_7_NO_2_)·0.42(C_6_H_8_N^+^·C_7_H_6_NO_2_^−^)
*M*_r_	230.3
Crystal system, space group	Monoclinic, *P* *c*
Temperature (K)	295
*a*, *b*, *c* (Å)	7.5970 (7), 11.6665 (12), 7.6754 (8)
β (°)	114.200 (3)
*V* (Å^3^)	620.49 (11)
*Z*	2
Radiation type	Mo *K*α
μ (mm^−1^)	0.09
Crystal size (mm)	0.28 × 0.24 × 0.20

Data collection	
Diffractometer	Bruker Kappa APEXII CCD
Absorption correction	Multi-scan (*SADABS*; Krause *et al.*, 2015 ▸)
*T*_min_, *T*_max_	0.977, 0.983
No. of measured, independent and observed [*I* > 3σ(*I*)] reflections	10064, 2144, 1330
*R*_int_	0.030
(sin θ/λ)_max_ (Å^−1^)	0.632

Refinement	
*R*[*F* > 3σ(*F*)], *wR*(*F*), *S*	0.031, 0.067, 1.29
No. of reflections	2144
No. of parameters	162
H-atom treatment	H atoms treated by a mixture of independent and constrained refinement
Δρ_max_, Δρ_min_ (e Å^−3^)	0.08, −0.08
Absolute structure	826 of Friedel pairs used in the refinement

Computer programs: *APEX2* and *SAINT* (Bruker, 2000 ▸), *SHELXS97* (Sheldrick, 2008 ▸), *JANA2006* (Petříček *et al.*, 2014 ▸), *PLATON* (Spek, 2009 ▸) and *DIAMOND* (Brandenburg & Putz, 2005 ▸).

## supplementary crystallographic information

### Crystal data

**Table:**

0.58(C_6_H_7_N·C_7_H_7_NO_2_)·0.42(C_6_H_8_N^+^·C_7_H_6_NO_2_^−^)	*F*(000) = 244
*M**_r_* = 230.3	*D*_x_ = 1.233 Mg m^−^^3^
Monoclinic, *P**c*	Mo *K*α radiation, λ = 0.71073 Å
Hall symbol: P -2yc	Cell parameters from 2749 reflections
*a* = 7.5970 (7) Å	θ = 3.4–21.8°
*b* = 11.6665 (12) Å	µ = 0.09 mm^−^^1^
*c* = 7.6754 (8) Å	*T* = 295 K
β = 114.200 (3)°	Block, colourless
*V* = 620.49 (11) Å^3^	0.28 × 0.24 × 0.20 mm
*Z* = 2	

### Data collection

**Table:**

Bruker Kappa APEXII CCD diffractometer	2144 independent reflections
Radiation source: fine-focus sealed tube	1330 reflections with *I* > 3σ(*I*)
Graphite monochromator	*R*_int_ = 0.030
ω and φ scan	θ_max_ = 26.7°, θ_min_ = 3.4°
Absorption correction: multi-scan (*SADABS*; Krause *et al.*, 2015)	*h* = −9→8
*T*_min_ = 0.977, *T*_max_ = 0.983	*k* = −14→14
10064 measured reflections	*l* = −9→9

### Refinement

**Table:**

Refinement on *F*^2^	3 constraints
*R*[*F* > 3σ(*F*)] = 0.031	H atoms treated by a mixture of independent and constrained refinement
*wR*(*F*) = 0.067	Weighting scheme based on measured s.u.'s *w* = 1/(σ^2^(*I*) + 0.0004*I*^2^)
*S* = 1.29	(Δ/σ)_max_ = 0.035
2144 reflections	Δρ_max_ = 0.08 e Å^−^^3^
162 parameters	Δρ_min_ = −0.08 e Å^−^^3^
0 restraints	Absolute structure: 826 of Friedel pairs used in the refinement

### Fractional atomic coordinates and isotropic or equivalent isotropic displacement parameters (Å^2^)

**Table:**

	*x*	*y*	*z*	*U*_iso_*/*U*_eq_	Occ. (<1)
C1	0.3688 (3)	0.10370 (17)	0.7142 (3)	0.0567 (10)	
C2	0.4435 (3)	0.20303 (17)	0.6720 (3)	0.0579 (10)	
H2	0.367077	0.247318	0.567618	0.0694*	
C3	0.6269 (3)	0.23627 (16)	0.7816 (3)	0.0548 (10)	
H3	0.673456	0.303544	0.750858	0.0658*	
C4	0.7464 (3)	0.17333 (18)	0.9371 (3)	0.0495 (8)	
C5	0.6731 (3)	0.07378 (17)	0.9790 (3)	0.0590 (11)	
H5	0.75078	0.029552	1.08292	0.0708*	
C6	0.4893 (3)	0.03939 (18)	0.8710 (3)	0.0619 (11)	
H6	0.443281	−0.027955	0.902126	0.0743*	
C7	0.9412 (3)	0.21074 (18)	1.0589 (3)	0.0586 (11)	
C8	0.4371 (3)	0.45466 (19)	0.3179 (3)	0.0722 (12)	
H8	0.357015	0.51507	0.317318	0.0867*	
C9	0.6296 (3)	0.4649 (2)	0.4249 (3)	0.0692 (12)	
H9	0.677992	0.53111	0.495943	0.083*	
C10	0.7528 (3)	0.3785 (2)	0.4289 (3)	0.0634 (11)	
C11	0.6709 (3)	0.2837 (2)	0.3229 (3)	0.0713 (12)	
H11	0.748112	0.222191	0.321632	0.0856*	
C12	0.4763 (4)	0.2786 (2)	0.2188 (3)	0.0758 (13)	
H12	0.424395	0.212877	0.147656	0.0909*	
C13	0.9662 (3)	0.3868 (2)	0.5459 (4)	0.0953 (14)	
H13a	1.011616	0.460296	0.525995	0.143*	0.5
H13b	1.030779	0.32749	0.507907	0.143*	0.5
H13c	0.99267	0.377834	0.678664	0.143*	0.5
N1	0.1826 (3)	0.0714 (2)	0.6077 (3)	0.0816 (11)	
H1b	0.139 (4)	−0.003 (2)	0.635 (4)	0.0979*	
H1a	0.119 (4)	0.104 (2)	0.496 (4)	0.0979*	
N2	0.3581 (3)	0.36282 (17)	0.2143 (3)	0.0708 (9)	
O1	0.9925 (2)	0.30981 (13)	1.0111 (2)	0.0797 (7)	
O2	1.0511 (2)	0.15736 (13)	1.1982 (2)	0.0771 (7)	
H1x	1.131103	0.335566	1.084979	0.131 (10)*	0.58 (6)
H1y	0.211109	0.33646	0.138026	0.131 (10)*	0.42 (6)
H13d	1.03067	0.399497	0.463082	0.143*	0.5
H13e	0.992562	0.449433	0.634092	0.143*	0.5
H13f	1.011833	0.316689	0.615392	0.143*	0.5

### Atomic displacement parameters (Å^2^)

**Table:**

	*U*^11^	*U*^22^	*U*^33^	*U*^12^	*U*^13^	*U*^23^
C1	0.0553 (15)	0.0586 (14)	0.0525 (14)	−0.0083 (13)	0.0185 (12)	−0.0083 (13)
C2	0.0593 (15)	0.0554 (13)	0.0484 (14)	0.0003 (11)	0.0115 (12)	0.0089 (11)
C3	0.0577 (14)	0.0525 (12)	0.0501 (14)	−0.0059 (11)	0.0179 (12)	0.0043 (11)
C4	0.0520 (12)	0.0483 (11)	0.0419 (12)	0.0043 (11)	0.0128 (10)	0.0032 (11)
C5	0.0675 (16)	0.0517 (14)	0.0475 (15)	0.0003 (12)	0.0131 (12)	0.0050 (11)
C6	0.0771 (18)	0.0505 (12)	0.0573 (15)	−0.0072 (12)	0.0269 (13)	0.0066 (12)
C7	0.0565 (16)	0.0533 (13)	0.0587 (15)	0.0028 (12)	0.0161 (13)	−0.0033 (13)
C8	0.0642 (16)	0.0608 (15)	0.0795 (18)	0.0030 (13)	0.0172 (14)	0.0014 (14)
C9	0.0671 (17)	0.0606 (15)	0.0677 (18)	−0.0086 (13)	0.0153 (13)	−0.0042 (12)
C10	0.0580 (16)	0.0743 (16)	0.0565 (15)	−0.0046 (14)	0.0219 (12)	0.0051 (14)
C11	0.0654 (16)	0.0736 (16)	0.0778 (19)	0.0022 (13)	0.0324 (15)	−0.0068 (14)
C12	0.0759 (18)	0.0752 (17)	0.0718 (19)	−0.0131 (15)	0.0257 (15)	−0.0155 (14)
C13	0.0599 (16)	0.108 (2)	0.102 (2)	−0.0065 (14)	0.0170 (14)	0.0027 (19)
N1	0.0661 (15)	0.0866 (17)	0.0751 (16)	−0.0152 (12)	0.0116 (13)	0.0095 (13)
N2	0.0563 (12)	0.0707 (13)	0.0739 (14)	−0.0070 (12)	0.0150 (10)	−0.0029 (11)
O1	0.0619 (10)	0.0671 (10)	0.0852 (12)	−0.0117 (8)	0.0050 (8)	0.0129 (9)
O2	0.0678 (11)	0.0708 (9)	0.0650 (11)	0.0061 (8)	−0.0011 (9)	0.0100 (8)

### Geometric parameters (Å, º)

**Table:**

C1—C2	1.386 (3)	C10—C13	1.500 (3)
C1—C6	1.395 (3)	C11—H11	0.9299
C1—N1	1.365 (3)	C11—C12	1.363 (3)
C2—H2	0.93	C12—H12	0.93
C2—C3	1.356 (3)	C12—N2	1.322 (3)
C3—H3	0.93	C13—H13a	0.96
C3—C4	1.378 (3)	C13—H13b	0.9599
C4—C5	1.382 (3)	C13—H13c	0.96
C4—C7	1.456 (3)	C13—H13d	0.96
C5—H5	0.93	C13—H13e	0.96
C5—C6	1.360 (3)	C13—H13f	0.96
C6—H6	0.93	N1—H1b	0.99 (3)
C7—O1	1.319 (3)	N1—H1a	0.88 (2)
C7—O2	1.223 (2)	H1b—H1a	1.61 (4)
C8—H8	0.9301	N2—H1x^i^	1.6303 (18)
C8—C9	1.357 (3)	N2—H1y	1.0719 (18)
C8—N2	1.322 (3)	O1—H1x	1.0154 (14)
C9—H9	0.93	O1—H1y^ii^	1.5740 (14)
C9—C10	1.367 (3)	H1x—H1y^ii^	0.5766 (1)
C10—C11	1.362 (3)		
			
C2—C1—C6	117.69 (18)	C10—C11—H11	119.8
C2—C1—N1	120.94 (18)	C10—C11—C12	120.4 (2)
C6—C1—N1	121.4 (2)	H11—C11—C12	119.79
C1—C2—H2	119.68	C11—C12—H12	118.53
C1—C2—C3	120.63 (17)	C11—C12—N2	123.0 (2)
H2—C2—C3	119.69	H12—C12—N2	118.52
C2—C3—H3	119.02	C10—C13—H13a	109.47
C2—C3—C4	122.0 (2)	C10—C13—H13b	109.47
H3—C3—C4	119.02	C10—C13—H13c	109.47
C3—C4—C5	117.65 (18)	C10—C13—H13d	109.47
C3—C4—C7	122.1 (2)	C10—C13—H13e	109.47
C5—C4—C7	120.19 (17)	C10—C13—H13f	109.47
C4—C5—H5	119.43	H13a—C13—H13b	109.48
C4—C5—C6	121.16 (18)	H13a—C13—H13c	109.47
H5—C5—C6	119.42	H13b—C13—H13c	109.47
C1—C6—C5	120.9 (2)	H13d—C13—H13e	109.48
C1—C6—H6	119.54	H13d—C13—H13f	109.47
C5—C6—H6	119.54	H13e—C13—H13f	109.47
C4—C7—O1	114.98 (17)	C1—N1—H1b	118.1 (13)
C4—C7—O2	124.0 (2)	C1—N1—H1a	119.1 (18)
O1—C7—O2	121.05 (18)	H1b—N1—H1a	120 (2)
H8—C8—C9	118.46	C8—N2—C12	116.79 (19)
H8—C8—N2	118.46	C8—N2—H1x^i^	129.06 (19)
C9—C8—N2	123.1 (2)	C8—N2—H1y	132.5 (2)
C8—C9—H9	119.78	C12—N2—H1x^i^	114.12 (16)
C8—C9—C10	120.4 (2)	C12—N2—H1y	110.21 (19)
H9—C9—C10	119.79	C7—O1—H1x	117.45 (14)
C9—C10—C11	116.3 (2)	N2^ii^—H1x—O1	173.65 (11)
C9—C10—C13	121.8 (2)	N2—H1y—O1^i^	173.55 (12)
C11—C10—C13	121.8 (2)		

Symmetry codes: (i) *x*−1, *y*, *z*−1; (ii) *x*+1, *y*, *z*+1.

### Hydrogen-bond geometry (Å, º)

**Table:**

*D*—H···*A*	*D*—H	H···*A*	*D*···*A*	*D*—H···*A*
N1—H1*b*···O2^iii^	0.99 (3)	2.04 (3)	3.028 (3)	179 (2)
N1—H1*a*···O2^i^	0.88 (2)	2.22 (3)	3.051 (3)	158 (3)
O1—H1*x*···N2^ii^	1.0154 (14)	1.6303 (18)	2.642 (2)	173.65 (11)
N2—H1*y*···O1^i^	1.0719 (18)	1.5740 (14)	2.642 (2)	173.55 (12)

Symmetry codes: (i) *x*−1, *y*, *z*−1; (ii) *x*+1, *y*, *z*+1; (iii) *x*−1, −*y*, *z*−1/2.
